# Gastrointestinal Endogenous Protein-Derived Bioactive Peptides: An *in Vitro* Study of Their Gut Modulatory Potential

**DOI:** 10.3390/ijms17040482

**Published:** 2016-04-01

**Authors:** Lakshmi A. Dave, Maria Hayes, Leticia Mora, Carlos A. Montoya, Paul J. Moughan, Shane M. Rutherfurd

**Affiliations:** 1 The Riddet Institute, Massey University, Private Bag 11222, Palmerston North 4442, New Zealand; P.Acharya@massey.ac.nz (L.A.D.); C.Montoya@massey.ac.nz (C.A.M.); P.J.Moughan@massey.ac.nz (P.J.M.); 2 Teagasc, The Irish Agricultural and Food Development Authority, Food BioSciences Department, Ashtown, Dublin 15, Ireland; Maria.Hayes@teagasc.ie; 3 Instituto de Agroquímica y Tecnología de Alimentos (CSIC), Avenida Agustín Escardino 7, 46980 Paterna, Valencia 46002, Spain; lemoso@iata.csic.es

**Keywords:** gut non-dietary proteins, lysozyme, serum albumin, angiotensin-I converting enzyme (ACE-I) inhibition, renin inhibition, dipeptidyl peptidase IV inhibition, antioxidant peptides

## Abstract

A recently proposed paradigm suggests that, like their dietary counterparts, digestion of gastrointestinal endogenous proteins (GEP) may also produce bioactive peptides. With an aim to test this hypothesis, *in vitro* digests of four GEP namely; trypsin (TRYP), lysozyme (LYS), mucin (MUC), serum albumin (SA) and a dietary protein chicken albumin (CA) were screened for their angiotensin-I converting (ACE-I), renin, platelet-activating factor-acetylhydrolase (PAF-AH) and dipeptidyl peptidase-IV inhibitory (DPP-IV) and antioxidant potential following simulated *in vitro* gastrointestinal digestion. Further, the resultant small intestinal digests were enriched to obtain peptides between 3–10 kDa in size. All *in vitro* digests of the four GEP were found to inhibit ACE-I compared to the positive control captopril when assayed at a concentration of 1 mg/mL, while the LYS < 3-kDa permeate fraction inhibited renin by 40% (±1.79%). The LYS < 10-kDa fraction inhibited PAF-AH by 39% (±4.34%), and the SA < 3-kDa fraction inhibited DPP-IV by 45% (±1.24%). The MUC < 3-kDa fraction had an ABTS-inhibition antioxidant activity of 150 (±24.79) µM trolox equivalent and the LYS < 10-kDa fraction inhibited 2,2-Diphenyl-1-picrylhydrazyl (DPPH) by 54% (±1.62%). Moreover, over 190 peptide-sequences were identified from the bioactive GEP fractions. The findings of the present study indicate that GEP are a significant source of bioactive peptides which may influence gut function.

## 1. Introduction

Gastrointestinal endogenous proteins (GEP) have been proposed [1,2] and recently identified as a potential source of bioactive peptides based on *in silico* analysis [3]. GEP are made up of gastrointestinal tract (GIT) epithelial turnover and gut microflora proteins [4] as well as soluble secreted proteins. These include the human mucins, digestive enzymes, and serum albumin [4]. Other contributors to GEP include the digestive hormones, immunoglobulins, lysozyme, and other gastric and intestinal peptides [5]. Previous *in silico* experiments [3] identified 25 GEP as potential sources of bioactive peptides possessing a range of biological activities. The aim of the present study was to investigate if the GEP trypsin (P00761 (TRYP)), human lysozyme (P61626 (LYS)), salivary mucin (P12021 (MUC)), human serum albumin (P02768 (SA)) and the dietary protein chicken albumin (P01012 (CA)) are precursor proteins for bioactive peptides which can be released following GI digestion. The selected proteins were screened for *in vitro* angiotensin-I converting enzyme (ACE-I; EC 3.4.15.1), renin (EC 3.4.23.15), platelet-activating factor-acetylhydrolase (PAF-AH; EC 3.1.1.47) and dipeptidyl peptidase-IV (DPP-IV; EC 3.4.14.5) inhibitory activities and *in vitro* antioxidant activities using the 2,2′-azino-bis(3-ethylbenzothiazoline-6-sulphonic acid) (ABTS) and 2,2-diphenyl-1-picrylhydrazyl (DPPH) inhibition assays. The selected proteins were digested using an *in vitro* gastrointestinal digestion model developed previously as part of the EU COST INFOGEST network [6]. After sequential *in vitro* gastric and small intestinal digestion, freeze-dried samples were assessed for their *in vitro* enzyme inhibitory activities.

Inhibition of the enzymes ACE-I, renin, PAF-AH and DPP-IV is known to lower systemic and local blood pressure, and assist in the alleviation of symptoms of several disorders including diabetes mellitus [7], hypercholesterolaemia, inflammatory diseases [8,9], and fibrosis [10]. Inhibition of ACE-I prevents the formation of angiotensin II, a potent vasoconstrictor while renin inhibition prevents the formation of angiotensin I, the precursor of angiotensin II [11]. It is now known that the GIT also contains a local renin angiotensin aldosterone system (RAAS) [12], which plays a role in intestinal fluid and electrolyte balance, and intestinal ischaemia [13]. PAF-AH catalyzes platelet-activating factor (PAF), a pro-inflammatory phospholipid mediator that is involved in various inflammatory diseases of the GIT [14], and elevated levels of PAF-AH are believed to be a risk factor for coronary heart disease [15] and systemic inflammation [16,17]. DPP-IV degrades the incretins including Glucagon-like peptide-2 (GLP-2). GLP-2 is known to help mucosal epithelial cell proliferation in the small intestine, and thus inhibition of DPP-IV in the GIT may enhance epithelial re-growth in the small intestine [18]. DPP-IV inhibition is also known to alleviate the symptoms of hypertension and diabetes mellitus [19] and also plays a role in regulation of satiety [20].

The lumen of the GIT is continually exposed to various pro-oxidants from the diet and the environment, and is thus the site of a significant amount of oxidative reactions [21]. Natural GEP-derived antioxidants could play a protective role against oxidative damage in the lumen. Therefore, the present study not only investigated the *in vitro* potential of GEP as a source of bioactive peptides with inhibitory activities against ACE-I, renin, DPP-IV and PAF-AH but also the antioxidant potential of these peptides.

## 2. Results

### 2.1. Digestion of Proteins and Determination of Protein Content Using Sodium Dodecyl Sulphate Polyacrylamide Gel Electrophoresis SDS-PAGE

**Figure 1:**
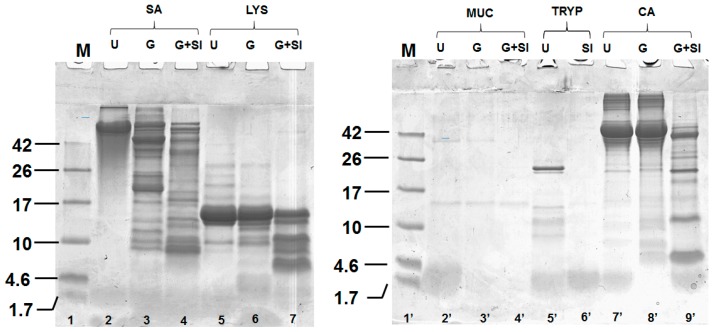
Tris-tricine SDS-PAGE, of the *in vitro* gastric and small intestinal digests of the gastrointestinal endogenous proteins, porcine trypsin (TRYP), human lysozyme (LYS), porcine salivary mucin (MUC) and human serum albumin (SA), and dietary protein chicken albumin (CA) conducted under reducing conditions. Each protein was analyzed prior to hydrolysis (U) and after gastric (G) and small intestinal digestion (G + SI).

LYS, MUC, SA, CA were subjected to simulated *in vitro* gastric and small intestinal digestion, and TRYP was subjected to simulated *in vitro* small intestinal digestion alone. The protein content of the gastric and small intestinal digests are given in Table 1. Tris-tricine sodium dodecyl sulphate polyacrylamide gel electrophoresis (SDS-PAGE) analysis was used to confirm the digestion of selected proteins (Figure 1). All of the proteins except LYS were digested extensively by the end of the simulated small intestinal phase of digestion as shown in Figure 1 lanes 2, 3 and 4 for SA, lanes 2′, 3′ and 4′ for MUC and lanes 5′ and 6′ for TRYP. Lanes 7′, 8′ and 9′ show the digestion of CA, which was used as a control (a known dietary source of bioactive peptides). In the case of LYS (lanes 5, 6 and 7), a significant amount of LYS (≈15 kDa band) remained intact even after sequential gastric and small intestinal digestion.

### 2.2. Angiotensin-I Converting Enzyme (ACE-I) Inhibition by Digests and Enriched Fractions

**Figure 2:**
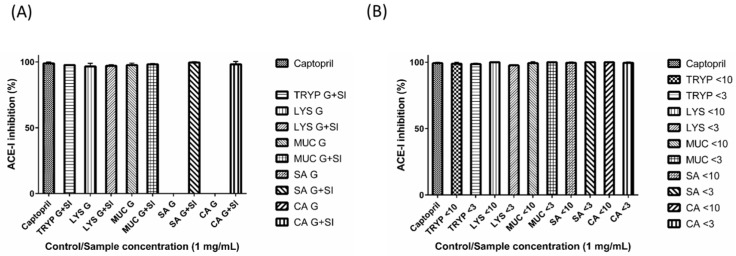
Angiotensin I converting enzyme (ACE-I) inhibition by (**A**) gastric (G) and small intestinal digests (G + SI) of the gastrointestinal endogenous proteins, porcine trypsin (TRYP), human lysozyme (LYS), porcine salivary mucin (MUC) and human serum albumin (SA), and dietary protein chicken albumin (CA); and (**B**) <10- and <3-kDa fractions of the small intestinal digests. All of the samples and the positive control captopril were tested at the concentration of 1 mg/mL.

The *in vitro* small intestinal digest of TRYP, and gastric and small intestinal digests of LYS, MUC, SA and CA were found to inhibit ACE-I significantly (≥ 97% inhibition); while the fractions TRYP < 10-kDa, TRYP < 3-kDa, LYS < 10-kDa, LYS < 3-kDa, MUC < 10-kDa, MUC < 3-kDa, SA < 10-kDa, SA < 3-kDa, CA < 10-kDa, CA < 3-kDa, inhibited ACE-I by 99% (±1.00%), 99% (±0.57%), 100% (±0.01%), 98% (±0.57%), 99% (±1.15%), 100% (±0.06%), 100% (±0.57%), 100% (±0.05%), 100% (±0), and 100% (±0.58%), respectively at a concentration of 1 mg/mL (Figure 2). These values were found to be comparable to the ACE-I inhibition values obtained for the positive control captopril (99% (±0.57%)) which was also assayed at a concentration of 1 mg/mL. To ensure that the observed ACE-I inhibition was due to the peptides present in the digested GEP fractions, rather than to the presence of the salts or other potentially interfering digestion medium constituents, all samples were desalted using dialysis as described previously. Dialyzed samples showed inhibition comparable to captopril when assayed at 1 mg/mL.

### 2.3. Renin Inhibition by Digests and Enriched Fractions

**Figure 3:**
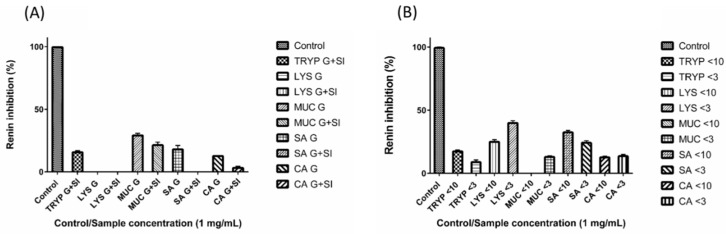
Renin inhibition by (**A**) gastric (G) and small intestinal digests (G + SI) of the gastrointestinal endogenous proteins, porcine trypsin (TRYP), human lysozyme (LYS), porcine salivary mucin (MUC) and human serum albumin (SA), and dietary protein chicken albumin (CA); and (**B**) <10- and <3- kDa fractions of the small intestinal digests. All of the samples were tested at the concentration of 1 mg/mL. The positive control Z-Arg-Arg-Pro-Phe-His-Sta-Ile-His-Lys-(Boc)-Ome was tested at 10 µM.

Among all the tested digests and fractions, the LYS < 3-kDa permeate fraction inhibited renin to the greatest extent (40% ±1.79 inhibition) (Figure 3). The small intestinal enriched fractions TRYP < 10-kDa, TRYP < 3-kDa, LYS < 10-kDa, MUC < 3-kDa, SA < 10-kDa, SA < 3-kDa, CA < 10-kDa and CA < 3-kDa inhibited renin by 17% (±1.19%), 9% (±1.70%), 25% (±1.76%), 13% (±0.93%), 32% (±1.51%), 24% (±1.64%), 13% (±0.82%), and 14% (±1.41%) respectively at a concentration of 1 mg/mL. TRYP SI, MUC G, MUC G + SI, SA G, and CA G were found to inhibit renin by 16% (±1.20%), 29% (±.68%), 21% (±2.39%), 18% (±3.17%), and 13% (±0.29%) respectively at a concentration of 1 mg/mL. From the gastric and small intestinal digests tested for renin inhibition LYS G, LYS G + SI, SA G + SI and CA G + SI were found to have negligible renin inhibitory potential. The fraction MUC < 10-kDa also showed no renin inhibition. The positive control (Z-Arg-Arg-Pro-Phe-His-Sta-Ile-His-Lys-(Boc)-Ome), showed 100% (±0.39%) inhibition when assayed at a concentration of 10 µM.

### 2.4. Renin, Platelet-Activating Factor-Acetylhydrolase (PAF-AH) Inhibition by Digests and Enriched Fractions

**Figure 4:**
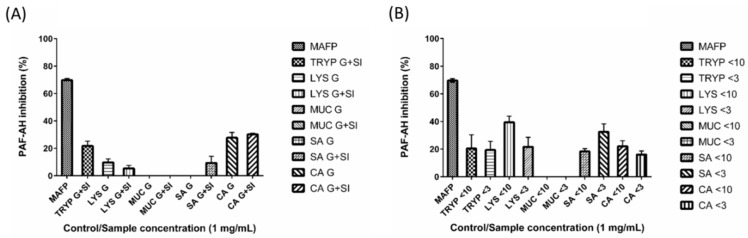
Platelet-activating factor-acetylhydrolase (PAF-AH) inhibition by (**A**) gastric (G) and small intestinal digests (G + SI) of the gastrointestinal endogenous proteins, porcine trypsin (TRYP), human lysozyme (LYS), porcine salivary mucin (MUC) and human serum albumin (SA), and dietary protein chicken albumin (CA); and (**B**) <10- and <3-kDa fractions of the small intestinal digests. All of the samples were tested at the concentration of 1 mg/mL. The positive control Methyl arachidonyl fluorophosphonate (MAFP) was tested at 260 ng/mL.

LYS < 10-kDa was found to inhibit PAF-AH by up to 39% (±4.34%), while the fractions TRYP < 10-kDa, TRYP < 3-kDa, LYS < 3-kDa, SA < 10-kDa, SA < 3-kDa, CA < 10-kDa, CA < 3-kDa showed 20% (±9.93%), 19% (±6.25%), 21% (±6.9%), 18% (±1.99%), 33% (±5.69%), and 22% (±4.01%) and 16% (±2.56%)) inhibition respectively at a concentration of 1 mg/mL (Figure 4). The fractions MUC < 10 and MUC < 3 showed no renin inhibition. TRYP SI, LYS G, LYS G + SI, SA G + SI, CA G and CA G + SI inhibited PAF-AH by 22% (±3.50%), 10% (±2.50%), 5% (±2.20%), 9% (±4.79%), 28% (±3.88%), and 30% (±0.81%) respectively at a concentration of 1 mg/mL. The digests MUC G, MUC G + SI and SA G showed no renin inhibition. The positive control MAFP inhibited PAF-AH by 70% (±1.31%).

### 2.5. Dipeptidyl Peptidase-IV Inhibitory (DPP-IV) Inhibition by Digests and Enriched Fractions

**Figure 5:**
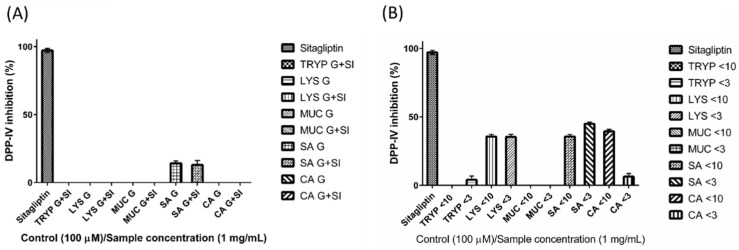
Dipeptidyl peptidase IV (DPP-IV) inhibition by (**A**) gastric (G) and small intestinal digests (G + SI) of the gastrointestinal endogenous proteins, porcine trypsin (TRYP), human lysozyme (LYS), porcine salivary mucin (MUC) and human serum albumin (SA), and dietary protein chicken albumin (CA); and (**B**) <10- and <3-kDa fractions of the small intestinal digests. All of the samples were tested at the concentration of 1 mg/mL. The positive control sitagliptin was tested at 100 µM.

The fractions LYS < 10-kDa, LYS < 3-kDa, SA < 10-kDa, SA < 3-kDa, and CA < 10-kDa inhibited DPP-IV by 36% (±1.52%), 35% (±1.93%), 36% (±1.52%), 45% (±1.24%), and 39% (±1.54%)respectively when assayed at a concentration of 1 mg/mL (Figure 5). The positive control Sitagliptin inhibited DPP-IV by 97% when assayed at the same concentration (±1.52%).

### 2.6. 2,2′-Azino-bis-3-ethylbenzthiazoline-6-sulphonic Acid Total Antioxidant Capacity (ABTS-TAC) of Digests and Enriched Fractions

**Figure 6:**
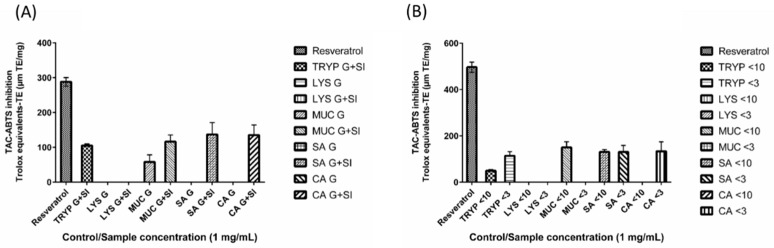
Total antioxidant capacity by 2,2′-azino-bis(3-ethylbenzothiazoline-6-sulphonic acid) (ABTS) inhibition by (**A**) gastric (G) and small intestinal digests (G + SI) of the gastrointestinal endogenous proteins, porcine trypsin (TRYP), human lysozyme (LYS), porcine salivary mucin (MUC) and human serum albumin (SA), and dietary protein chicken albumin (CA); and (**B**) <10- and <3-kDa fractions of the small intestinal digests. All of the samples and the positive control resveratrol were tested at the concentration of 1 mg/mL and the results are expressed in µM trolox equivalents (TE).

Among all of the tested digests and fractions, the fraction MUC < 10-kDa quenched the ABTS radical maximally and had an 2,2′-azino-bis-3-ethylbenzthiazoline-6-sulphonic acid total antioxidant capacity (ABTS-TAC) value of 150 (± 24.79) µM·TE/mg; while the-fractions TRYP < 10-kDa, TYRP < 3-kDa, SA < 10-kDa, SA < 3-kDa AND CA < 3-kDa had ABTS-TAC values of 50 (± 4.91), 114 (± 17.81), 131 (± 10.54), 131 (± 28.09) and 134 (± 40.84) µM TE (Figure 6). The *in vitro* digests TRYP SI, MUC G, MUC G + SI, SA G + SI, and CA G + SI were found to have ABTS-TAC of 105 (± 4.62), 58 (± 20.57), 116 (± 19.12), 137 (± 34.27) and 135 (± 29.32) µM·TE/mg. The digests LYS G, LYS G + SI, SA G and CA G were found to have no antioxidant potential using the ABTS-TAC assay. Fractions LYS < 10-kDa, LYS < 3-kDa, MUC < 3-kDa and CA < 10-kDa did not inhibit/quench the ABTS radical. The positive control resveratrol was found to have an ABTS-TAC values ranging from 288 to 497 (±12.38–22.11) TE/mg.

### 2.7. DPPH Inhibition by Digests and Enriched Fractions

**Figure 7:**
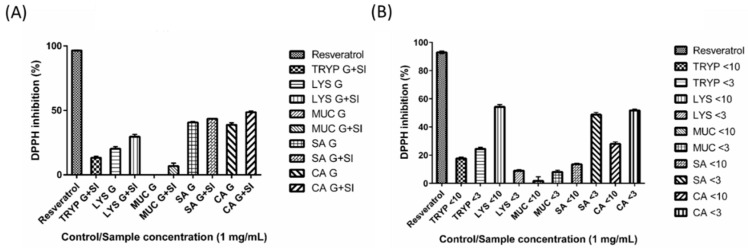
2,2-diphenyl-1-picrylhydrazyl (DPPH) inhibition by (**A**) gastric (G) and small intestinal digests (G + SI) of the gastrointestinal endogenous proteins, porcine trypsin (TRYP), human lysozyme (LYS), porcine salivary mucin (MUC) and human serum albumin (SA), and dietary protein chicken albumin (CA); and (**B**) <10- and <3-kDa fractions of the small intestinal digests. All of the samples and the positive control resveratrol were tested at the concentration of 1 mg/mL.

The fractions LYS < 10-kDa, CA < 3-kDa, SA < 3-kDa, were found to scavenge the DPPH radical by 54% (±1.62%), 52% (±0.89%) and 49% (±1.58%), respectively at a concentration of 1 mg/mL (Figure 7). The remaining enriched fractions (TRYP < 10-kDa, TRYP < 3-kDa, LYS < 3-kDa, MUC < 10-kDa, MUC < 3-kDa, SA < 10-kDa, CA < 10-kDa) inhibited the DPPH radical by 18% (±1.12%), 24% (±1.17%), 9% (±0.85%), 2% (±3.03%), 8% (±1.32%), 14% (±0.69%), 28% (±1.35%) respectively at a concentration of 1 mg/mL. The un-fractionated digests TRYP SI, LYS G, LYS G + SI, SA G, SA G + SI, CA G, CA G + SI inhibited the DPPH free radical by 13% (±1.15%), 20% (±1.68%), 30% (±1.84%), 41% (±0.69%), 44% (±0.24%), 39% (±1.59%) and 49% (±1.00%) respectively at a concentration of 1 mg/mL. The MUC G digest showed no DPPH inhibition, while the MUC G + SI digest inhibited DPPH by 7% (±2.29%). The positive control, resveratrol, inhibited DPPH by 93%–97% when assayed at the same concentration (±0.95%–0.36%).

### 2.8. Electrospray Ionisation Time of Flight Mass Spectrometry (ESI-TOF-MS) Characterisation of Peptides

A total of 19 and 91 peptides were identified in the <3-kDa fraction of the small intestinal digests of lysozyme and serum albumin respectively and 13 and 70 peptides were identified from the <10-kDa fraction of the small intestinal digests of lysozyme and serum albumin respectively (Table 2). The peptides ranged from 7–36 amino acids in chain length.

## 3. Discussion

To date several studies have investigated the quantity of GEP found in the digestive contents of animals [4,22,23] and humans [24,25,26], and the effect of dietary constituents such as protein (and its structure), fiber and anti-nutritional factors on the GEP [27,28,29,30]. It is now widely accepted that GEP contribute significantly to the amino acid pool in the GIT, and consequently are now recognized as important in addition to estimation of the true amino acid requirements of humans [5,31]. However, in spite of the knowledge that up to 80% of the GEP secreted into the gut lumen are digested and reabsorbed by the end of the terminal ileum, the possibility that they may also give rise to bioactive peptides has only recently been considered [1]. Quantitatively, in mammals, GEP are secreted in an equal or greater amount as dietary proteins ingested per day [26]. However, while an extensive range of dietary proteins have been investigated for their ability to generate bioactive peptides during food processing, fermentation and gastrointestinal digestion *in vivo*, GEP have not been widely investigated. Therefore, analogous to enzymatically-derived dietary bioactive peptides, peptides derived from GEP in the gut lumen may also possess gut-specific bioactivities [2].

The present study used an *in vitro* digestion model to examine GEP as a source of peptides with physiological effects including heart-health regulatory, GIT health beneficial, anti-inflammatory and weight gain prevention. Four GEP and one dietary protein (chicken albumin) were selected to examine their potential to generation bioactive peptides following simulated GIT digestion. This work demonstrates that *in vitro* gastric and small intestinal digests of GEP possess biological activity relevant to the GIT and overall human health. These biological activities include potential renin, ACE-I, DPP-IV inhibitory and antioxidant activities.

GEP used in this work included TRYP, LYS, MUC, and SA. These were subjected to simulated, sequential gastric and small intestinal digestion using pepsin and pancreatin (containing proteases from the exocrine cells of the porcine pancreas), while TRYP was only subjected to small intestinal digestion. Pepsin and proteolytic enzymes present in pancreatin digested all of the four GEP as shown using SDS-PAGE (Figure 1), and the extent of digestion generally compared favorably to the protein CA (Figure 1). LYS was not completely digested and this finding is in agreement with a previous study that demonstrated that hen egg lysozyme, which is 57.5% homologous (BLAST identity match) to human lysozyme [32], was only partially digested in the GIT [33].

All of the gastric and small intestinal digests, and the enriched fractions containing peptides <10- and <3-kDa in size inhibited ACE-I when assayed at a concentration of 1 mg/mL and results obtained were comparable to ACE-I inhibition values obtained for captopril and for food derived ACE-I inhibitory peptides identified previously. To ensure that the observed ACE-I inhibition was from the peptides in the tested GEP digests rather than the salts and chemical constituents of the *in vitro* digestion medium, the samples were desalted by subjecting them to dialysis, and the dialyzed samples also showed inhibition comparable to captopril (data not shown). In comparison, dietary bioactive peptides, from various sources including cereals, legumes, meat, fish and poultry, have been documented to show a wide range of ACE-I inhibition ranging from 1% to ≥90% inhibition [34,35,36]. The ACE-I inhibition activity observed suggests that the enzyme may be inhibited by a broad-spectrum of peptidic inhibitors. From a gut perspective, inhibition of ACE-I is important since it is now known that an extensive RAAS exists within the GIT. While the GIT-RAAS is known to balance the fluid and electrolyte dynamics in the gut, it is not entirely clear as to what the exact role of ACE-I inhibitors may be in the context of the gut. It is important to note that, postprandial or functional hyperemia (an increased blood flow to the gut after a meal (postprandial state)) is a known phenomenon that helps in the digestion of food [37,38], and it may be that the dietary protein- and GEP-derived ACE-I inhibitory peptides play a protective role against a severe rise in local hypertension in the GIT.

Only a few GEP digests including TRYP SI, MUC G, MUC G + SI, SA G and CA G inhibited renin. While the LYS G + SI digest did not inhibit renin the fractions LYS <10-kDa and <3-kDa were found to inhibit renin by 25% and 40% each, indicating that low molecular weight peptides derived from LYS may be responsible for the observed inhibition. In comparison to previously reported values for renin and PAF-AH inhibition by peptides from dietary proteins, inhibition values obtained in the present work were comparable. For example, a previous study investigated the renin inhibition of a papain digest of the red seaweed *Palmaria palmata* and reported that a reverse phase high pressure liquid chromatography (RP-HPLC) fraction of the latter hydrolysate inhibited renin by 58.97% (±1.26%) when assayed at a concentration of 1 mg/mL [39].

Although the small intestinal digest of LYS and SA inhibited PAF-AH by 5% and 9%, their enriched fractions (LYS < 10-kDa and SA < 3-kDa) inhibited the PAF-AH by 39% and 33% respectively. This suggests that smaller peptides derived from LYS and SA may be more effective inhibitors of PAF-AH. The PAF-AH inhibitory values obtained in this work were comparable to those obtained previously for a papain hydrolysate of *Palmaria palmata* which inhibited PAF-AH by 30.37% [40].

A similar trend was observed in the case of DPP-IV inhibition by GEP subjected to GI digestion. While the gastric and small intestinal digests of all of the proteins showed either no inhibition or low inhibition values (SA G and SA G + SI), following enrichment of the small intestinal digests a substantial increase in the DPP-IV inhibitory activity was observed. For example, the SA < 3-kDa, SA < 10-kDa, LYS < 10-kDa and LYS < 3-kDa were found to inhibit DPP-IV by greater than 35% at a concentration of 1 mg/mL. In particular, the DPP-IV inhibition exerted by SA < 3-kDa (45% (±1.24%)), is comparable to the DPP-IV inhibition reported for the gastrointestinal digests of oat flour, alcalase digest of oat glutelin and a tryptic digest of highland barley glutelin that were found to have IC_50_ values of 0.99, 0.13 and 1.83 mg/mL respectively [41]. In relation to gut health, the optimal inhibition of DPP-IV may aid in remediation of non-steroidal anti-inflammatory drug-induced intestinal ulcers [42], by possibly enhancing the beneficial amino acid- and bile acid-induced bicarbonate secretion in the small intestine [43], besides promoting epithelial regrowth [18] and stimulating intestinal growth [44].

The extent of inhibition of the ACE-I, renin, DPP-IV and PAF-AH enzymes observed in the current work is comparable to that of various food protein digests [40,45,46,47]. The inhibition of all of the above mentioned enzymes by peptides is associated with a positive impact on cardiovascular health and reduction of high blood pressure [19,48,49]. However, ACE-I, renin, DPP-IV and PAF-AH are enzymes that are also present in the gut, and recent evidence suggests that inhibition of these enzymes can impact positively on gut health and may help to prevent necrosis, ulcerative colitis and other inflammatory disorders of the human gut [8,9,13,17].

Furthermore, the digested GEP and enriched fractions were also found to have considerable antioxidant activity *in vitro*. Among the digests that were able to inhibit ABTS ion (TRYP SI, MUC G, MUC G + SI, SA G + SI, and CA G + SI), SA G + SI had the highest total antioxidant capacity value (137 µM·TE/mg), which is much higher than the previously reported values (0.47 µM·TE/mg protein) for the 3-kDa fraction of *in vitro* digests of human milk [50]. However, the highest TAC-ABTS values obtained for GEP in the present study are significantly lower than the values for human plasma (430–569 µM·TE) which was analyzed for TAC-ABTS using ABTS and hydrogen peroxide [51] previously. In addition, a small increase in the ABTS scavenging was observed after fraction enrichment, particularly for MUC protein. However, in the antioxidant potential measurement using the DPPH radical assay, we found a greater difference between the un-fractionated GEP digest fractions and the enriched fraction of the same GEP. For example, LYS G + SI inhibited DPPH by only 30% (±1.84%) but the <10-kDa fraction of the same sample inhibited DPPH by 54% (±1.62), followed by the SA <3-kDa that also inhibited DPPH by 49% (±1.58%). The antioxidant activity of GEP-derived peptides may be of great importance to gut health as an exclusive dependence on diet-derived antioxidants may not be entirely possible given that our diet varies considerably [1]. The gut undergoes a significant turnover of the epithelial lining and is constantly exposed to the external environment and various oxidants on a daily basis, thus GEP may form an intrinsic source of potential antioxidant peptides that can offset this regular damage.

In this work, the GEP serum albumin and lysozyme were found to inhibit enzymes in all of the *in vitro* bioassays carried out and the results were comparable to enzyme inhibition values obtained for the dietary protein chicken albumin (CA). In particular, the <10- and <3-kDa fractions of SA and LYS were found to be the most active. Consequently, the latter two fractions were examined further using ESI-TOF MS to characterize the peptides present. Thirty-two peptides were identified in the enriched fractions (10- and 3-kDa) of LYS, and 161 different peptides in the SA fractions. Most of the identified peptides were within the general range of the chain length reported for bioactive peptides (3–30 amino acids) [52,53], but tending towards the longer chain length (7–36 amino acids). Overall, among all of the 22 amino acids, LYS has the highest amounts of the amino acids A (10.8%), R (10.8%) and N (7.7%). In terms of the most commonly found amino acids in bioactive peptide sequences, LYS sequences contained significant amounts of A (10.8%), R (10.8%), G (8.5%), L (6.2%), V (6.9%), Q (4.6%) and Y (4.6%). In the <3 and <10 kDa fractions of LYS, the major amino acids observed were A, V, N, Q and Y. Most of the peptide fractions identified were from the f(62–130) region of the protein, and a few from the f(27–47) region of the protein. In particular, from the LYS fractions that showed the highest DPPH inhibition, we have identified 10 peptide sequences that have an N residue at the N-terminal of the peptide. These results are in agreement with the findings of Rajapakse, *et al.* [54], who identified the two peptides NADFGLNGLEGLA and NGLEGLK, containing an N-terminal N residue. These peptides, generated from squid, showed the greatest antioxidant effect in an *in vitro* analysis based on lipid peroxidation studies previously. Overall, the SA protein derived peptides contained the highest amounts (up to 10%) of the amino acids A, E, L, T and K. In terms of the most commonly found amino acids in bioactive peptide sequences, SA was found to be rich in L (10.4%), V (7%), F (5.3%), T (4.8%), P (4.1%) and R (4.1%). We observed the highest frequency of occurrence of peptides with D, E, K, F, L, V, A, R, T and S in the peptides identified in the <3- and <10-kDa fraction of SA. Within the <3- and <10-kDa fraction of SA, over 80 peptide fragments with a hydrophobic or aromatic amino acid residues of A, V, L and F were identified, and the presence of these peptides is known to have a positive impact on the renin inhibition [55] and antioxidant potential of peptides [56,57,58]. Thus, while both the LYS and SA fractions were found to contain peptides with previously identified bioactive amino acids such as A, L, V, R, T and F [54,59,60], the observed range of bioactivity in this study suggests that apart from the constituting amino acids, the peptide structure and certain other amino acids may also play a role in the bioactivity of various hydrolysates.

A majority of the peptides identified in the present study are novel sequences that have not been reported from these GEP previously. However, we found two peptides in the LYS < 3-kDa fractions (DPQGIRAWV (f(120–128)), and PQGIRAWVAW (f(121–130))) that are subsequences [61] of a previously identified antimicrobial sequence reported from human lysozyme DNIADAVACAKRVVRDPQGIRAWVAWRNR (f(87–115)) [35,62]. Future work will specifically aim to chemically synthesize the identified peptides and examine their individual biological activities, which will allow us to determine which particular GEP-derived peptide sequences result in ACE-I, renin, DPP-IV, PAF-AH and antioxidant activity.

The present study, although limited in its scope, due to its *in vitro* nature, is the first to illustrate that GEP are a source bioactive peptides with *in vitro* ACE-I, renin, PAF-AH and DPP-IV inhibitory and antioxidant activities. The known amino acid composition of the peptides identified in the present work may have contributed to the observed biological activities *in vitro*. However, the peptide structure and certain lesser studied amino acids that were also found to be present in abundance may also have positively contributed to the observed *in vitro* bioactivities. Indeed, some of the food derived bioactive peptides with IC_50_ values in the range of 100 to 500 µM can be of nutritive/physiological importance in that they can be active after oral administration [63]. It is known that peptides greater than 4 amino acids in size are usually not absorbed into the blood. However, the bioavailability of the peptides in this work should be further studied using *in vitro* transcytosis assays using cell models with epithelial cell lines including the human epithelial cororectal adenocarcinoma cells (Caco-2) or human brain microvascular endothelial cell lines (hCMEC/D3) as previously reported [64]. Further *in vivo* work is essential to determine if GEP can indeed give rise to bioactive peptides during their digestion in the gastrointestinal tract. However, the present study points towards the possibility that the GEP may have the potential to constitute a feedback-like peptidergic system within the gut lumen, particularly considering that the GEP are constantly present in the gut and can be modulated by dietary constituents.

## 4. Experimental Section

### 4.1. Materials and Reagents

Human recombinant LYS (P61626), human recombinant SA (P02768), porcine TRYP (P00761), porcine MUC (Apomucin; (P12021)), CA (P01012), dimethyl sulfoxide, porcine salivary amylase, porcine pepsin and porcine pancreatin, the antioxidant resveratrol and the ACE-I inhibitor captopril, 2,2-diphenyl-1-picrylhydrazyl (DPPH), and all of the tris-tricine SDS-PAGE reagents were supplied by Sigma Aldrich (Dublin, Ireland). The ACE-I inhibition assay kit was supplied by Dojindo EU, GmbH (Kumamoto, Japan), the 2,2′-azino-bis(3-ethylbenzothiazoline-6-sulphonic acid) (ABTS) inhibition based total antioxidant capacity assay kit was supplied by BioVision Inc. (Milpitas, CA, USA), the ABTS total antioxidant capacity assay kit was supplied by Zen-Bio, Inc. (Research Triangle Park, NC, USA) and the renin, PAF-AH and DPP-IV inhibition assay kits were all supplied by the Cayman Chemical Company (Cambridge BioSciences, Cambridge, UK). The molecular weight cut-off filters were obtained from Millipore (Tullagreen, Carrigtwohill, Cork city, Ireland). The 3.5 kDa dialysis membrane was supplied by the medical supply company (Medical supply company (MSC), Dublin, Ireland). All other chemicals used were of analytical grade (≥99% purity). Unless otherwise stated, all reagents were made using Milli-Q deionized water.

### 4.2. Simulated Gastrointestinal Digestion of Selected Gastrointestinal Endogenous Proteins (GEP) and a Food Protein Comparator

Four secreted endogenous gut proteins were selected for the present study, namely, porcine trypsin (TRYP), human recombinant lysozyme (LYS), porcine salivary mucin (MUC) and serum albumin (SA). Chicken albumin (CA) was included in the study for the purpose of comparison, as it is a known source of food-derived bioactive peptides [65,66,67,68]. The molecular weights and the chain lengths of the proteins examined in the present study are presented in Table 3.

**Figure 8:**
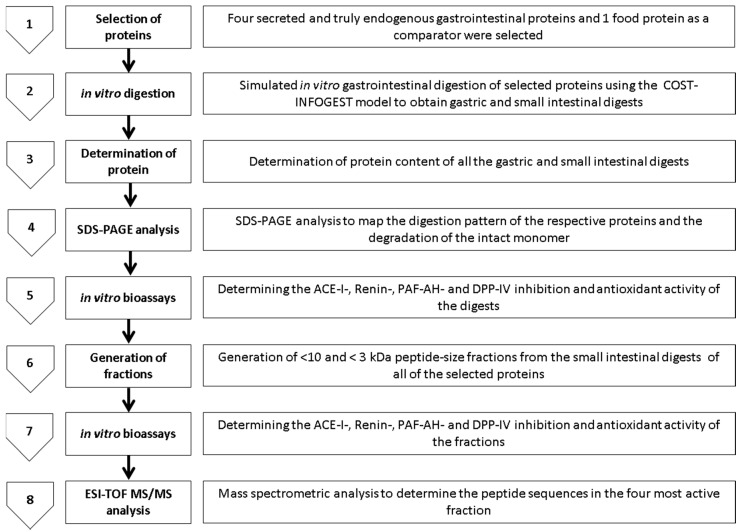
The *in vitro* experimental design and methodologies used to study the angiotensin I converting enzyme (ACE-I), renin, Platelet-activating factor-acetylhydrolase (PAF-AH) and dipeptidyl peptidase IV (DPP-IV) inhibition, and Total antioxidant capacity by 2,2′-azino-bis(3-ethylbenzothiazoline-6-sulphonic acid) (ABTS) and 2,2-diphenyl-1-picrylhydrazyl (DPPH) inhibition based antioxidant activity of the gastric and small intestinal hydrolysates and the small intestinal <10- and <3-kDA fractions of gastrointestinal endogenous proteins porcine trypsin, porcine salivary mucin, human recombinant lysozyme and serum albumin, and food protein chicken albumin. SDS-PAGE: Tris tricine sodium dodecyl sulphate polyacrylamide gel electrophoresis; ESI-TOF MS/MS: Electrospray ionization time of flight mass spectrometry.

All of the studied proteins were subjected to simulated static *in vitro* gastrointestinal digestion using the INFOGEST method described previously [6]. Selected proteins were subjected to sequential gastric and small intestinal digestions with the exception of TRYP. TRYP was subjected only to small intestinal digestion as it enters the GIT lumen at the duodenum [69]. The simulated *in vitro* sequential digestion was performed in triplicate. Samples (0.5 mL × 3) of the hydrolysates of the chosen GEP at a concentration of 0.5–0.0125 g/mL were collected after the gastric phase (G) and small intestinal phase (G + SI) of digestion, freeze-dried and stored at −30 °C until further analysis. The overall methodology and major steps followed in the present study are shown in Figure 8. Furthermore, MWCO-based ultra-filtration was applied to all of the small intestinal *in vitro* digests, to obtain <10- and <3-kDa sized peptide-enriched fractions (labelled as TRYP/LYS/MUC/SA/CA <3- and <-10 kDa respectively). All of the samples were desalted using a 3.5 kDa dialysis membrane.

### 4.3. Total Protein Analysis

The total protein content was determined using the Dumas method in accordance with the AOAC method 992.15 (1990) and using a LECO FP628 Protein analyzer (LECO Corp., St. Joseph, MI, USA). A conversion factor of 6.25 was used to estimate the protein content from the nitrogen content.

### 4.4. Tris Tricine Sodium Dodecyl Sulphate Polyacrylamide Gel Electrophoresis (SDS-PAGE) Analysis

Tris tricine SDS-PAGE peptide analysis was carried out using previously described methods [70,71]. GEP protein samples were diluted 1:10 with reducing PAGE sample buffer (2% or 4% SDS and 100 mM dithiothreitol (DTT)) to obtain a concentration of 1 mg/mL. 5–10 µL of each diluted sample was loaded onto a SDS-PAGE resolving gel (49.5% T, 3% C). The components of the resolving and stacking gels are given in supplementary Table S1.

The loaded gel was run in a Bio-Rad Laboratories Miniprotean gel apparatus at a voltage of 100 V for 2 h or until the dye front approached the bottom of the gel. Gels were stained using Coomassie brilliant blue R250 (0.3%) for 1 h at 20 °C. Destaining was carried out using 10% *v*/*v* acetic acid and 10% *v*/*v* 2-propanol. Gels were destained for 48 h at 20 °C, with gentle shaking. Gels were scanned on a molecular Gel Doc XR system (Bio-Rad Laboratories, Berkeley, CA, USA) and the gel images were analyzed using QuantityOne and ImageLab software programs (Bio-Rad Laboratories, Berkeley, CA, USA).

### 4.5. ACE-I Inhibition Assay

This assay was carried out using an ACE-I inhibitor assay kit in accordance with the manufacturer’s instructions. All fractions were assayed at a concentration of 1 mg/mL sample dissolved in HPLC grade water in triplicate. The known ACE-I inhibitor Captopril was used as a positive control at a concentration of 1 mg/mL. ACE-I inhibition percentage values for each peptide were calculated using the absorbance values obtained at 450 nm and the equation:

% ACE-I inhibition = (Absorbance of Blank 1 − Absorbance value of the Inhibitor)/(Absorbance of Blank 1 − Blank 2) × 100

where, blank 1 is control without the addition of any inhibitor, blank 2 is the reagent blank, and the inhibitor is the positive control or test sample (protein digest/peptide fraction).

### 4.6. Renin Inhibition Assay

The renin inhibition assay was carried out using a renin inhibitor screening kit in accordance with the manufacturer’s instructions. Z-Arg-Arg-Pro-Phe-His-Sta-Ile-His-Lys-(Boc)-Ome (Sigma Aldrich, Dublin, Ireland), a known renin inhibitor, was used as a positive control at a concentration of 10 µM. The % inhibition of renin was calculated using the fluorescence values obtained and the following formula:

% renin inhibition = (100% Initial activity values (AF) − fluorescence value obtained for the test sample)/100% initial activity (AF) × 100

where, test sample is protein digest/peptide fraction.

### 4.7. PAF-AH Inhibition Assay

This assay was performed using a PAF-AH inhibitor kit supplied by Cambridge BioSciences, Cambridge, UK in accordance with the manufacturer’s instructions. All of the digests and enriched fractions were assessed in triplicate. Methyl arachidonyl fluorophosphonate (MAFP), a known PAF-AH inhibitor was used as a positive control at a concentration of 260 ng/mL.

### 4.8. DPP-IV Inhibition

The DPP-IV inhibitory activities of the digested protein samples were determined using a DPP-IV inhibitor assay kit (Cambridge BioSciences, Cambridge, UK) in accordance with the manufacturer’s instructions. Sitagliptin, a known DPP-IV inhibitor, was used as a positive control at a concentration of 100 µM. The percentage inhibition of DPP-IV was calculated as follows:

% Inhibition of DPP-IV = (RFU DPP-IV activity − RFU Inhibitor)/(RFU DPP−IV activity) × 100

where, RFU DPP-IV activity is the fluorescence (measured in relative fluorescence units (RFU)) of the measured without addition of any inhibitor, and RFU Inhibitor is the RFU measured in the presence of the Sitagliptin or the test digests or enriched fractions.

### 4.9. 2,2′-Azino-bis(3-ethylbenzothiazoline-6-sulphonic acid) (ABTS)-Based Total Antioxidant Capacity (ABTS-TAC) Assay

The assay was carried out using an ABTS antioxidant assay kit and performed as described by the manufacturer. Trolox was used to obtain a standard curve and the assay buffer was used as a blank. Resveratrol was used as a positive control. Antioxidant activity was calculated using the trolox standard curve and expressed as µM trolox equivalents (TE).

### 4.10. DPPH Inhibition

The DPPH inhibition assay was performed in accordance with previously described methods [72,73]. Briefly, triplicates of the digest or enriched fraction samples (1 mg/mL) and the positive control (resveratrol) were prepared in the assay buffer. The samples and positive control solutions (285 µL) were each combined with a freshly prepared methanolic DPPH solution in a Nunc™ 96-well microWell™ plate to obtain a final DPPH concentration of 100 µM. The plate was incubated in dark at room temperature for 30 min. The absorbance was then read at 515 nm. The control reaction mixture contained DPPH and methanol without sample. The percentage DPPH inhibition was calculated using the following equation:

% Inhibition DPPH = (Abs Control − Abs Inhibitor)/Abs Control × 100

where, Abs Control is the absorbance of the DPPH solution without any resveratrol or test peptides and Abs Inhibitor is the absorbance of the test samples or positive control.

### 4.11. Electrospray Ionization Time of Flight Mass Spectrometry (ESI-TOF MS) Characterisation of Peptides Present in the Most Active Fractions

The four fractions which showed the greatest extent of renin, DPP-IV, PAF-AH inhibition and antioxidant activities were selected for peptide characterization. Briefly, the nano LC-MS/MS analysis was performed using an Eksigent Nano-LC Ultra 1D Plus system (Eksigent of AB Sciex, Framingham, CA, USA) coupled to the quadrupole-time-of-flight (Q-ToF) TripleTOF^®^ 5600 system from AB Sciex Instruments (Framingham, MA, USA) that was equipped with a nano-electrospray ionization source.

Lyophilized samples were re-suspended in 500 µL of H_2_O with 0.1% of TFA, and 20 µL of each sample at different times of processing were cleaned and concentrated using Zip-Tip C18 with standard bed format (Millipore Corporation, Bedford, MA, USA) according to manufacturer’s guidelines, and kept at −20 °C until analysis. Then, 5 µL of the supernatant was injected into the LC-MS/MS system.

After 5 min of pre-concentration, the trap column was automatically switched in-line onto a nano-HPLC capillary column (3 µm, 75 µm × 12.3 cm, C18) (Nikkyo Technos Co, Ltd., Tokyo, Japan). Mobile phase A contained 0.1% *v*/*v* formic acid in water, and mobile phase B, contained 0.1% *v*/*v* FA in 100% acetonitrile. A linear gradient from 5%–35% of solvent B over 90 min, and 10 min from 35%–65% of solvent B, at a flow rate of 0.3 μL/min and a column temperature of 30 °C was used. The column outlet was directly coupled to a nano-electrospray ion (nESI) source. Operating conditions for the Q/ToF mass spectrometer were positive polarity and information-dependent acquisition (IDA) mode was used. A 0.25-s ToF MS scan from *m*/*z* of 100 to 1200 was performed, followed by 0.05-s product ion scans from *m*/*z* of 100 to 1500 on the 50 most intense 1+ to 5+ charged ions. Automated spectral processing, peak list generation, database search, and *de novo* sequencing for the identification of peptides were performed using Mascot Distiller v2.4.2.0 software (Matrix Science, Inc., Boston, MA, USA) (hppt://www.matrixscience.com). The UniProt protein database was used to identify the peptides with a significance threshold *p* < 0.1 and a False Discovery Rate of 1.5%. The taxonomy used for identification in the database was *homo sapiens*. The tolerance on the mass measurement was 0.3 Da in MS mode and MS/MS ions.

## Figures and Tables

**Table 1:** The protein content of the *in vitro* gastric (G) and small intestinal digests (G + SI) of the gastrointestinal endogenous proteins, porcine trypsin (TRYP), human lysozyme (LYS), porcine salivary mucin (MUC) and human serum albumin (SA), and dietary protein chicken albumin (CA).

Hydrolysate	Protein Content (%)
TRYP G + SI	16.38
LYS G	97.89
LYS G + SI	98.48
MUC G	45.35
MUC G + SI	42.89
SA G	90.12
SA G + SI	87.71
CA G	85.56
CA G + SI	82.26

**Table 2:** Gastrointestinal endogenous proteins (GEP)-derived peptide sequences found in the <3- and <10-kDa fractions of the *in vitro* small intestinal digests of lysozyme and serum albumin. The samples were analyzed using electrospray ionization time of flight mass spectrometry (ESI-TOF MS).

Parent Protein and Hydrolysate Fraction	Peptide Amino acid Sequence	Protein Fragment	Observed Mass (Da)	Theoretical Mass (Da)	Observed *m*/*z*	Theoretical *m*/*z*	Theoretical *Z*
Lysozyme < 3-kDa fraction	ALLQDNIADAV	f(101–111)	1141.60	1141.60	571.81	571.81	2
	ALLQDNIADAVA	f(101–112)	1212.64	1212.64	607.33	607.32	2
	ARTLKRLGMDGYRGISL	f(27–43)	1906.06	1906.06	477.52	477.52	4
	DPQGIRAWV	f(120–128)	1040.54	1040.54	521.28	521.28	2
	GIFQINSRYW ^a^	f(73–82)	1298.66	1298.64	650.34	650.33	2
	GMDGYRGISLANWM	f(34–47)	1569.71	1569.71	785.86	785.86	2
	LGMDGYRGISL	f(33–43)	1180.59	1180.59	591.30	591.30	2
	LGMDGYRGISLA	f(33–44)	1251.63	1251.63	626.82	626.82	2
	LLQDNIADAV ^b^	f(102–111)	1071.54	1071.54	536.78	536.78	2
	NAGDRSTDYG	f(64–73)	1054.43	1054.43	528.22	528.22	2
	NAGDRSTDYGIFQ	f(64–76)	1442.64	1442.64	722.33	722.33	2
	NAGDRSTDYGIFQI	f(64–77)	1555.73	1555.73	778.87	778.87	2
	NAGDRSTDYGIFQIN	f(64–78)	1669.77	1669.77	835.89	835.89	2
	NYNAGDRSTD	f(62–71)	1111.45	1111.45	556.73	556.73	2
	NYNAGDRSTDYGIF	f(62–75)	1591.69	1591.69	796.85	796.85	2
	NYNAGDRSTDYGIFQ	f(62–76)	1719.76	1719.75	860.89	860.88	2
	NYNAGDRSTDYGIFQI	f(62–77)	1832.84	1832.83	917.43	917.42	2
	PQGIRAWVAW	f(121–130)	1182.63	1182.63	592.32	592.32	2
	YNAGDRSTDYGIF	f(63–75)	1477.65	1477.65	739.83	739.83	2
Lysozyme < 10-kDa fraction	CNDGKTPGAV	f(83–92)	960.43	960.43	481.22	481.22	2
	CNDGKTPGAVNACHLSCS ^c,d^	f(83–100)	1773.73	1773.74	592.25	592.25	3
	GIFQINSRYW	f(73–82)	1282.65	1282.65	642.33	642.33	2
	GMDGYRGISLANWM	f(34–47)	1569.72	1569.71	785.87	785.86	2
	NAGDRSTDYG	f(64–73)	1054.43	1054.43	528.22	528.22	2
	NAGDRSTDYGIFQI	f(64–77)	1555.74	1555.73	778.88	778.87	2
	NYNAGDRSTD	f(62–71)	1111.45	1111.45	556.73	556.73	2
	NYNAGDRSTDY	f(62–72)	1274.52	1274.52	638.27	638.27	2
	NYNAGDRSTDYG	f(62–73)	1331.53	1331.54	666.77	666.78	2
	NYNAGDRSTDYGIFQI	f(62–77)	1832.85	1832.83	917.43	917.42	2
	SALLQDNIADAV	f(100–111)	1228.63	1228.63	615.32	615.32	2
	YNAGDRSTDYG	f(63–73)	1217.49	1217.49	609.75	609.75	2
	ALLQDNIADAVACA ^e^	f(101–114)	1434.66	1434.67	718.34	718.34	2
Serum albumin < 3-kDa fraction	AEAKDVFLGMFL	f(344–355)	1339.69	1339.68	1339.69	670.85	2
	AEFAEVSKLVTDL	f(250–262)	1420.75	1420.74	1420.75	711.38	2
	AEFAEVSKLVTDLT ^f^	f(250–263)	1549.79	1549.79	1549.79	775.90	2
	AEFAEVSKLVTDLTK	f(250–264)	1649.89	1649.89	1649.89	825.95	2
	AEFAEVSKLVTDLTKVHT	f(250–267)	1987.10	1987.06	1987.10	994.54	2
	AEVENDEMPADLPSLA	f(315–330)	1699.76	1699.76	1699.76	850.89	2
	AEVSKLVTDLT	f(253–63)	1174.64	1174.64	1174.64	588.33	2
	AKVFDEFKPL	f(395–404)	1192.65	1192.65	1192.65	597.33	2
	AKVFDEFKPLVEEPQ	f(395–409)	1774.91	1774.91	1774.91	888.46	2
	ALEVDETYVPK	f(514–524)	1262.64	1262.64	1262.64	632.33	2
	ALEVDETYVPKE	f(514–525)	1391.68	1391.68	1391.68	696.85	2
	ALEVDETYVPKEF	f(514–526)	1538.75	1538.75	1538.75	770.38	2
	ALVLIAFA	f(45–52)	816.51	816.51	816.51	409.26	2
	ALVLIAFAQ	f(45–53)	944.57	944.57	944.57	473.29	2
	ALVLIAFAQY	f(45–54)	1107.62	1107.63	1107.62	554.82	2
	AVMDDFAAFVEK	f(570–581)	1341.63	1341.63	1341.63	671.82	2
	DDNPNLPR	f(131–138)	939.44	939.44	939.44	470.73	2
	DEFKPLVEEPQNLI	f(399–412)	1669.86	1669.86	1669.86	835.94	2
	DEFKPLVEEPQNLIK	f(399–413)	1797.95	1797.95	1797.95	899.98	2
	DETYVPKE	f(518–525)	979.45	979.45	979.45	490.73	2
	DLGEENFKALV	f(37–47)	1233.63	1233.62	1233.63	617.82	2
	DLGEENFKALVL	f(37–48)	1346.71	1346.71	1346.71	674.36	2
	DLPSLAADFVES	f(325–336)	1262.60	1262.60	1262.60	632.31	2
	DLPSLAADFVESK	f(325–337)	1390.70	1390.70	1390.70	696.36	2
	DVFLGMFLYE ^g^	f(348–357)	1248.77	1248.57	1248.77	625.29	2
	EDHVKLVNEVTEFA	f(61–74)	1628.81	1628.80	1628.81	815.41	2
	ENDEMPADLPSL	f(318–329)	1329.58	1329.58	1329.58	665.80	2
	ENDEMPADLPSLAADFVES	f(318–336)	2048.90	2048.89	2048.90	1025.45	2
	ENDEMPADLPSLAADFVESK	f(318–337)	2177.01	2176.98	2177.01	1089.50	2
	EQLKAVMDD	f(566–574)	1047.49	1047.49	1047.49	524.75	2
	EQLKAVMDDF ^h^	f(566–575)	1176.55	1176.55	1176.55	589.28	2
	EQLKAVMDDFA ^h^	f(566–576)	1247.59	1247.59	1247.59	624.80	2
	EQLKAVMDDFAA ^h^	f(566–577)	1318.62	1318.62	1318.62	660.32	2
	EVDETYVPK	f(516–524)	1078.52	1078.52	1078.52	540.27	2
	EVDETYVPKE	f(516–525)	1207.57	1207.56	1207.57	604.79	2
	EVDETYVPKEF	f(516–526)	1354.63	1354.63	1354.63	678.32	2
	EVDETYVPKEFN	f(516–527)	1468.67	1468.67	1468.67	735.34	2
	EVDETYVPKEFNAET ^i^	f(516–530)	1770.79	1770.78	1770.79	886.40	2
	FDEFKPLVEEPQNLIK	f(398–413)	1945.02	1945.02	1945.02	973.52	2
	FKDLGEEN	f(35–42)	950.44	950.43	950.44	476.22	2
	FKDLGEENFKALV	f(35–47)	1508.79	1508.79	1508.79	755.40	2
	FKDLGEENFKALVL	f(35–48)	1621.87	1621.87	1621.87	811.94	2
	FPKAEFAEVSKLVTDLT	f(247–263)	1894.01	1894.01	1894.01	632.34	3
	FYAPELLFFAK	f(173–183)	1344.71	1344.71	1344.71	673.36	2
	KPLVEEPQN	f(402–410)	1052.55	1052.55	1052.55	527.28	2
	KPLVEEPQNLI	f(402–412)	1278.72	1278.72	1278.72	640.37	2
	KVPQVSTPT	f(438–446)	955.53	955.53	955.53	478.77	2
	KVPQVSTPTLV	f(438–448)	1167.69	1167.69	1167.69	584.85	2
	KVPQVSTPTLVEV	f(438–450)	1395.79	1395.80	1395.79	698.91	2
	KVPQVSTPTLVEVS	f(438–451)	1482.83	1482.83	1482.83	742.42	2
	LDELRDEG	f(206–213)	945.44	945.44	945.44	473.73	2
	LEVDETYVPK	f(515–524)	1191.60	1191.60	1191.60	596.81	2
	LEVDETYVPKE	f(515–525)	1320.65	1320.64	1320.65	661.33	2
	LQHKDDNPNLPR	f(127–138)	1445.73	1445.74	1445.73	482.92	3
	LVAASQAALG	f(599–608)	899.51	899.51	899.51	450.76	2
	LVNEVTEFA	f(66–74)	1020.52	1020.51	1020.52	511.26	2
	LVNEVTEFAK	f(66–75)	1148.61	1148.61	1148.61	575.31	2
	LVNEVTEFAKTCVADESAENCDK ^c,d^	f(66–88)	2512.11	2512.13	2512.11	629.04	4
	LVRPEVDVM ^g^	f(139–147)	1072.56	1072.56	1072.56	537.29	2
	NDEMPADLPSLA ^i^	f(318–330)	1272.56	1272.55	1272.56	637.28	2
	NDEMPADLPSLAADF	f(318–333)	1604.71	1604.70	1604.71	803.36	2
	NDEMPADLPSLAADFVES	f(318–336)	1919.86	1919.85	1919.86	960.93	2
	NDEMPADLPSLAADFVESK	f(318–337)	2047.95	2047.94	2047.95	1024.98	2
	NEVTEFAKTCVADESAENCDK ^c,d^	f(68–88)	2299.96	2299.98	2299.96	1150.99	2
	NRRPCFSALEVDETYVPKE ^d,j^	f(507–525)	2199.96	2200.09	2199.96	734.37	3
	NYAEAKDVFLG	f(342–352)	1225.60	1225.60	1225.60	613.81	2
	NYAEAKDVFLGM	f(342–353)	1356.64	1356.64	1356.64	679.33	2
	NYAEAKDVFLGMFL	f(342–355)	1616.83	1616.79	1616.83	809.40	2
	QHKDDNPNLPR ^h^	f(128–138)	1315.62	1315.63	1315.62	439.55	3
	QYLQQCPFEDHV ^d^	f(53–64)	1471.68	1471.67	1471.68	736.84	2
	RETYGEM	f(105–111)	884.37	884.37	884.37	443.19	2
	RHPDYSVV	f(361–368)	971.48	971.48	971.48	486.75	2
	RHPDYSVVL	f(361–369)	1084.57	1084.57	1084.57	543.29	2
	SALEVDETYVPK	f(513–524)	1349.67	1349.67	1349.67	675.84	2
	SALEVDETYVPKE	f(513–525)	1478.72	1478.71	1478.72	740.36	2
	SALEVDETYVPKEF	f(513–526)	1625.79	1625.78	1625.79	813.90	2
	TPVSDRVT	f(491–498)	873.45	873.46	873.45	437.74	2
	VFDEFKP	f(397–403)	880.43	880.43	880.43	441.22	2
	VFDEFKPL	f(397–404)	993.52	993.52	993.52	497.77	2
	VFDEFKPLVEEPQ	f(397–409)	1575.78	1575.78	1575.78	788.90	2
	VFDEFKPLVEEPQN	f(397–410)	1689.83	1689.82	1689.83	845.92	2
	VFDEFKPLVEEPQNLI	f(397–412)	1916.00	1915.99	1916.00	959.00	2
	VKLVNEVTEFA	f(64–74)	1247.68	1247.68	1247.68	624.85	2
	VLIAFAQYL	f(47–55)	1036.60	1036.60	1036.60	519.31	2
	VPQVSTPTLVEVS	f(439–451)	1354.74	1354.73	1354.74	678.37	2
	VSTPTLVEVS	f(442–451)	1030.56	1030.55	1030.56	516.28	2
	DVFLGMFL ^g^	f(348–355)	956.47	956.47	956.47	479.24	2
	TLRETYGEMADCCAKQEPERNECFLQHKDDNPNLPR ^d,i^	f(103–138)	4216.84	4216.90	4216.84	844.39	5
	LVNEVTEFAKTC ^d^	f(66–77)	1318.71	1318.68	1318.71	660.35	2
	ENDEMPADLPSLA ^h^	f(318–330)	1382.60	1382.60	1382.60	692.31	2
	LSVVLNQLCVL ^c^	f(477–87)	1231.69	1231.68	1231.69	616.85	2
Serum albumin < 10-kDa fraction	AEAKDVFLGMF	f(344–354)	1226.60	1226.60	614.31	614.31	2
	AEAKDVFLGMFL	f(344–355)	1339.70	1339.68	670.86	670.85	2
	AEFAEVSKLVTDL	f(250–262)	1420.76	1420.74	711.39	711.38	2
	AEFAEVSKLVTDLT	f(250–263)	1521.91	1521.79	761.96	761.90	2
	AEFAEVSKLVTDLTK	f(250–264)	1649.88	1649.89	550.97	550.97	3
	AEVSKLVTDLT	f(253–263)	1174.65	1174.64	588.33	588.33	2
	AKVFDEFKPLVEEPQNLIK	f(395–413)	2243.25	2243.22	748.76	748.75	3
	ALVLIAFAQ	f(45–53)	944.56	944.57	473.29	473.29	2
	ALVLIAFAQY	f(45–54)	1107.63	1107.63	554.82	554.82	2
	APELLFFAK	f(175–183)	1034.57	1034.58	518.29	518.30	2
	AVMDDFAAFVE	f(570–580)	1213.54	1213.53	607.78	607.77	2
	AVMDDFAAFVEK	f(570–581)	1341.64	1341.63	671.83	671.82	2
	DEFKPLVEEPQNL	f(399–411)	1556.80	1556.77	779.41	779.39	2
	DEFKPLVEEPQNLI	f(399–412)	1669.89	1669.86	835.95	835.94	2
	DEFKPLVEEPQNLIK	f(399–413)	1798.00	1797.95	900.01	899.98	2
	DLGEENFKALVLI	f(37–49)	1459.82	1459.79	730.92	730.90	2
	DLGEENFKALVLIA	f(37–50)	1530.85	1530.83	766.43	766.42	2
	DLGEENFKALVLIAFAQ	f(37–53)	1877.06	1876.99	939.54	939.50	2
	DLPSLAADF	f(325–333)	947.45	947.46	474.73	474.74	2
	DLPSLAADFVES	f(325–334)	1262.61	1262.60	632.31	632.31	2
	DLPSLAADFVESK	f(325–335)	1390.72	1390.70	696.37	696.36	2
	DVFLGMFLY ^g^	f(348–366)	1119.53	1119.53	560.77	560.77	2
	DVFLGMFLYE ^g^	f(348–367)	1248.57	1248.57	625.29	625.29	2
	DVFLGMFLYEYA ^g^	f(348–369)	1482.70	1482.67	742.36	742.34	2
	ENDEMPADLPSLA	f(318–330)	1400.63	1400.61	701.32	701.31	2
	EQLKAVMDDF	f(566–575)	1176.55	1176.55	589.28	589.28	2
	EQLKAVMDDFAA	f(566–577)	1318.63	1318.62	660.32	660.32	2
	EQLKAVMDDFAAF	f(566–578)	1483.73	1483.70	742.87	742.86	2
	EVDETYVPKE	f(516–525)	1207.56	1207.56	604.79	604.79	2
	FDEFKPLVEEPQNLIK	f(398–413)	1945.04	1945.02	649.36	649.35	3
	FKDLGEENFKALV	f(35–47)	1508.81	1508.79	755.41	755.40	2
	FKDLGEENFKALVL	f(35–48)	1621.91	1621.87	811.96	811.94	2
	FKDLGEENFKALVLI	f(35–49)	1735.00	1734.96	868.51	868.49	2
	FKDLGEENFKALVLIA	f(35–50)	1806.04	1805.99	904.03	904.00	2
	FKDLGEENFKALVLIAFAQ	f(35–53)	2152.24	2152.16	1077.13	1077.09	2
	FPKAEFAEVSKLVTDLT	f(247–263)	1894.01	1894.01	632.35	632.34	3
	FYAPELLFFA	f(173–182)	1216.62	1216.62	609.32	609.32	2
	FYAPELLFFAK	f(173–183)	1344.73	1344.71	673.37	673.36	2
	FYAPELLFFAKR	f(173–184)	1500.79	1500.81	501.27	501.28	3
	KDLGEENFKALVLIAFAQ	f(36–53)	2005.11	2005.09	669.38	669.37	3
	KVPQVSTPT	f(438–446)	955.52	955.53	478.77	478.77	2
	KVPQVSTPTLVEV	f(438–450)	1395.81	1395.80	698.91	698.91	2
	KVPQVSTPTLVEVS	f(438–451)	1482.85	1482.83	742.43	742.42	2
	KVPQVSTPTLVEVSRN ^k^	f(438–453)	1710.00	1709.92	856.01	855.97	2
	LQHKDDNPNLPR	f(127–138)	1445.71	1445.74	482.91	482.92	3
	LVNEVTEFAKTCVADESAENCDKSLHTLF ^c,k^	f(66–94)	3210.60	3210.50	1071.21	1071.17	3
	NDEMPADLPSLA	f(319–330)	1271.58	1271.57	636.80	636.79	2
	NDEMPADLPSLAADFVESK	f(319–337)	2048.01	2047.94	1025.01	1024.98	2
	NYAEAKDVFLGMF	f(342–354)	1503.73	1503.71	752.87	752.86	2
	NYAEAKDVFLGMFL ^g^	f(342–355)	1632.81	1632.79	817.41	817.40	2
	RHPDYSVVLLL	f(361–371)	1310.74	1310.73	656.38	656.37	2
	SALEVDETYVPK	f(513–524)	1349.68	1349.67	675.85	675.84	2
	TKKVPQVSTPTLVEVS^f^	f(436–451)	1739.99	1739.97	871.00	870.99	2
	TYETTLEK	f(376–383)	983.47	983.48	492.74	492.75	2
	VFDEFKPL	f(397–404)	993.50	993.52	497.76	497.77	2
	VFDEFKPLVEEPQ	f(397–409)	1575.81	1575.78	788.91	788.90	2
	VFDEFKPLVEEPQN	f(397–410)	1689.86	1689.82	845.94	845.92	2
	VFDEFKPLVEEPQNL	f(397–411)	1802.96	1802.91	902.49	902.46	2
	VFDEFKPLVEEPQNLI	f(397–412)	1916.05	1915.99	959.03	959.00	2
	VFDEFKPLVEEPQNLIK	f(397–413)	2044.11	2044.09	682.38	682.37	3
	VFDEFKPLVEEPQNLIKQ	f(397–414)	2172.21	2172.15	725.08	725.06	3
	VLIAFAQYL	f(47–55)	1036.58	1036.60	519.30	519.31	2
	VPQVSTPTLVEV	f(439–450)	1267.71	1267.70	634.86	634.86	2
	VPQVSTPTLVEVS	f(439–451)	1354.75	1354.73	678.38	678.37	2
	EQLKAVMDDFA	f(566–576)	1265.60	1265.60	633.81	633.81	2
	YAPELLFFAK	f(174–183)	1197.65	1197.64	599.83	599.83	2
	LVRPEVDV	f(139–146)	925.51	925.52	463.76	463.77	2
	EVDETYVPK	f(516–524)	1078.51	1078.52	540.26	540.27	2
	LSQRFPKAEFAEVSKLVTDLT	f(243–263)	2379.28	2378.28	595.83	595.58	4
	VLIAFAQY	f(47–54)	923.49	923.51	462.75	462.76	2

^a^ Deamidated W; ^b^ Deamidated Q; ^c^ Dioxidation(C); ^d^ Cys– > Dha(C); ^e^ Trioxidation(C); ^f^ Formyl(K); ^g^ Oxidation(M); ^h^ Glu– > pyro-Glu@N-term; ^i^ Deamidated(N); ^j^ Dehydrated(S); ^k^ Arg– > GluSA(R).

**Table 3:** Gastrointestinal endogenous proteins (GEP) and dietary protein examined in the *in vitro* study.

Protein	Chain Length	Molecular Weight (*M*w) (KDa)	Uniprot Accession No.
Gut cryptome protein	–	–	–
Trypsin (TRYP)	223	24	P00761
Lysozyme (LYS)	148	17	P61626
Porcine salivary mucin (Apomucin) (MUC)	1150	110	P12021
Serum albumin (SA)	609	69	P02768
Dietary protein	–	–	–
Chicken albumin (CA)	385	43	P01012

## Supplementary Materials

Supplementary materials can be found at http://www.mdpi.com/1422-0067/17/4/482/s1.

## Abbreviations

ABTS	2,2′-azino-bis(3-ethylbenzothiazoline-6-sulphonic acid)
ACE-I	angiotensin-I Converting Enzyme
DPPH	2,2-diphenyl-1-picrylhydrazyl
DPP-IV	dipeptidyl peptidase-IV
GEP	Gastrointestinal endogenous proteins
GLP-2	Glucagon-like peptide-2
MAFP	Methyl arachidonyl fluorophosphonate
MWCO	Molecular weight cut-off
PAF-AH	platelet-activating factor-acetylhydrolase
RAAS	renin angiotensin aldosterone system
SDS-PAGE	sodium dodecyl sulphate polyacrylamide gel electrophoresis
