# Supplementary material for: Gastrointestinal Endogenous Protein-Derived Bioactive Peptides: An in Vitro Study of Their Gut Modulatory Potential
Source: Int J Mol Sci. 2016 Apr 1;17(4):482. doi: 10.3390/ijms17040482 (PMC4848938; doi:10.3390/ijms17040482)
Supplement: Supplementary file 1 [file ijms-17-00482-s001.pdf]

Supplementary Materials: Gastrointestinal Endogenous Protein-Derived Bioactive Peptides: An *in Vitro* Study of Their Gut Modulatory Potential

Lakshmi A. Dave, Maria Hayes, Leticia Mora, Carlos A. Montoya, Paul J. Moughan and Shane M. Rutherfurd

Table S1. Constituents of tris-tricine SDS-PAGE gel.

Constituents [a]	Resolving Gel	Stacking Gel
Concentration	16%	4%
Milli-Q water	2.96 mL	4.15 mL
3 M Tris-HCl buffer (pH 8.45)	3 mL	1.5 mL
Glycerol (90%)	1 g	–
Acrylamide (49.5%)	3.25 mL	0.5 mL
10% Ammonium persulfate	50 µL	50 µL
TEMED	5 µL	5 µL

[a] The quantities listed here are for the preparation of two gels.

Detailed Methodology of Bioassays

ACE-I Inhibition Assay

This assay was carried out using an ACE-I inhibitor assay kit in accordance with the manufacturer's instructions. All fractions were assayed at a concentration of 1 mg/mL sample dissolved in HPLC grade water in triplicate. Briefly, prior to carrying out the assay, the enzyme working solution was prepared by dissolving enzyme B, provided by the manufacturer, in 2.0 mL of deionized water and by adding 1.5 mL of this solution to enzyme A (provided by the manufacturer). The indicator working solution was prepared by dissolving the coenzyme and enzyme C, both provided by the manufacturer, with 3 mL of HPLC grade water each and adding 2.8 mL of each of them to the indicator solution. A negative control was prepared by adding 20 µL of HPLC grade water and 20 µL of substrate buffer. Reagent blank wells were prepared by adding 40 µL of HPLC grade water and 20 µL of substrate buffer. Inhibitor wells were prepared by adding 20 µL of sample and 20 µL of substrate buffer. The known ACE-I inhibitor Captopril was used as a positive control at a concentration of 1 mg/mL. The enzymatic reaction was started by adding 20 µL of enzyme working solution to each inhibitor and negative control wells. The microwell plate was covered and incubated for one hour at 37 °C. After 1 h, 200 µL of indicator working solution were added to each well and the microwell plate was further incubated at room temperature for ten minutes. Absorbance was measured with a FLUOstar Omega microplate reader (BMG LABTECH GmbH, Offenburg, Germany) at 450 nm. ACE-I inhibition percentage for each peptide was calculated using the equation:

$$\% \text{ Inhibition ACE-1} = (\text{Blank 1} - \text{Inhibitor})/(\text{Blank 1} - \text{Blank 2}) \times 100$$

where, Blank 1 is control without the addition of any inhibitor, Blank 2 is reagent blank, and inhibitor is positive control or test sample (protein digest/peptide fraction).

Renin Inhibition Assay

The renin inhibition assay was carried out using a renin inhibitor screening kit in accordance with the manufacturer's instructions. Briefly, the assay buffer and samples were incubated at 37 °C prior to assay. The assay was started by adding 20 µL of substrate, 160 µL of assay buffer and 10 µL of dimethyl sulfoxide to the background wells in the microplate. Next, 20 µL of substrate, 150 µL of assay buffer was added to the 100% initial activity wells. Following this, 20 µL of substrate, 150 µL of assay buffer, and 10 µL of the test samples was added to the test sample wells. The reaction was

initiated by the addition of the enzyme renin (10 μL) to all the wells except the background wells. To mix the reagents, the plate was placed on a shaker for 10 s and subsequently covered and incubated for 15 min at 37 °C. Z-Arg-Arg-Pro-Phe-His-Sta-Ile-His-Lys-(Boc)-Ome (Sigma Aldrich, Dublin, Ireland), a known renin inhibitor, was used as a positive control at a concentration of 10 μM. The % inhibition of renin was calculated using the following formula:

$$\% \text{ Inhibition} = \frac{100\% \text{ Initial Activity (AF)} - \text{Test Sample}}{100\% \text{ Initial Activity (AF)}} \times 100$$

where, test sample is protein digest/peptide fraction.

PAF-AH Inhibition Assay

This assay was performed using a PAF-AH inhibitor kit supplied by Cambridge BioSciences, UK and in accordance with the manufacturer's instructions. Briefly, the PAF-AH inhibitor assay buffer was prepared by diluting 3 mL of assay buffer stock with 27 mL of HPLC grade water to obtain a final concentration of 0.1 M Tris–HCl at pH 7.2. The substrate, 2-thio PAF was reconstituted in 12 mL of working assay buffer to a concentration of 400 μM. The background wells contained 10 μL of assay buffer, 200 μL of the substrate and 10 μL of solvent (working assay buffer). Initial activity wells were prepared by adding 200 μL of the substrate solution and 10 μL of the solvent. Inhibitor wells were prepared by adding 200 μL of the substrate solution and 10 μL of the test digests or enriched fractions. The reaction was initiated by the addition of 10 μL of human plasma PAF-AH to all of the wells excepting the background wells. Finally, the plate was covered and incubated for 20 min at 25 °C. Post incubation, 10 μL of 5,5′-dithiobis-(2-nitrobenzoic acid) was added to each well to complete the reaction. All of the digests and enriched fractions were assessed in triplicate. Methyl arachidonyl fluorophosphonate (MAFP), a known PAF-AH inhibitor was used as a positive control at a concentration of 260 ng/mL.

DPP-IV Inhibition

The DPP-IV inhibitory activities of the digested protein samples were determined using a DPP-IV inhibitor assay kit (Cambridge BioSciences, Cambridge, UK) in accordance with the manufacturer's instructions. Briefly, samples (1 mg/mL) were prepared in the assay buffer (20 mM Tris-HCL containing 100 mM NaCl and 1 mM EDTA, pH 8.0). A solution of human recombinant DPP-IV enzyme was prepared in assay buffer and kept on ice prior to use. The substrate solution contained 5 mM Gly-Pro-Aminomethylcoumarin (AMC) in assay buffer. Sitagliptin, a known DPP-IV inhibitor, was used as a positive control at a concentration of 100 μM. The test digests and enriched fractions and the positive control solution were incubated with the combined enzyme and substrate solutions in a covered microwell plate at 37 °C for 30 min. The fluorescence intensity of the liberated AMC was measured using a FLUOstar Omega microplate reader (BMG LABTECH Gmbh, Offenburg, Germany) with an excitation wavelength of 355 nm and an emission wavelength of 460 nm. The percentage inhibition of DPP-IV was calculated as follows:

$$\% \text{ Inhibition DPP} - \text{IV} = \frac{[\text{RFU DPP} - \text{IV activity} - \text{RFU Inhibitor}]}{\text{RFU DPP} - \text{IV activity}} \times 100$$

where, RFU DPP-IV activity is the fluorescence (measured in relative fluorescence units (RFU)) of the measured without addition of any inhibitor, and RFU Inhibitor is the RFU measured in the presence of the Sitagliptin or the test digests or enriched fractions.

2,2′-Azino-bis(3-ethylbenzothiazoline-6-sulphonic acid) (ABTS)-Based Total Antioxidant Capacity (ABTS-TAC) Assay

The assay was carried out using an ABTS antioxidant assay kit and performed as described by the manufacturer. Briefly, the working myoglobin solution was prepared by adding 25 μL of myoglobin stock solution to the provided 2.457 mL of dilution buffer. Trolox was used to obtain a standard curve and the assay buffer was used as a blank and resveratrol was used as a positive

control. Ten μL of standard, test sample, blank or positive control was added to the individual wells of a Nunc™ 96 microplate, followed by the addition of 20 μL of myoglobin working solution to the standard, samples and positive control. The assay was commenced by the addition of ABTS solution to all the wells. The plate was incubated at room temperature with shaking for 5 min. The reaction was immediately stopped using the provided stop solution and the plate was read at 405 nm. Antioxidant activity was calculated using the trolox standard curve and expressed as μM trolox equivalents (TE).
